# Non-Modifiable Risk Factors for Stress Fractures in Military Personnel Undergoing Training: A Systematic Review

**DOI:** 10.3390/ijerph19010422

**Published:** 2021-12-31

**Authors:** Grace M. Lennox, Patrick M. Wood, Ben Schram, Elisa F. D. Canetti, Vini Simas, Rodney Pope, Robin Orr

**Affiliations:** 1 Doctor of Physiotherapy Program, Bond University, Gold Coast, QLD 4226, Australia; grace.lennox@student.bond.edu.au (G.M.L.); patrick.wood@student.bond.edu.au (P.M.W.); ecanetti@bond.edu.au (E.F.D.C.); rpope@csu.edu.au (R.P.); rorr@bond.edu.au (R.O.); 2 Tactical Research Unit, Bond University, Gold Coast, QLD 4226, Australia; vsimas@bond.edu.au; 3 School of Community Health, Charles Sturt University, Albury-Wodonga, NSW 2640, Australia

**Keywords:** trainees, recruits, stress fracture, bone, army, navy, air-force, defence

## Abstract

A fracture, being an acquired rupture or break of the bone, is a significant and debilitating injury commonly seen among athletes and military personnel. Stress fractures, which have a repetitive stress aetiology, are highly prevalent among military populations, especially those undergoing training. The primary aim of this review is to identify non-modifiable risk factors for stress fractures in military personnel undergoing training. A systematic search was conducted of three major databases to identify studies that explored risk factors for stress fractures in military trainees. Critical appraisal, data extraction, and a narrative synthesis were conducted. Sixteen articles met the eligibility criteria for the study. Key non-modifiable risk factors identified were prior stress fracture and menstrual dysfunction, while advancing age and race other than black race may be a risk factor. To reduce the incidence of stress fractures in military trainees, mitigating modifiable risk factors among individuals with non-modifiable risk factors (e.g., optimising conditioning for older trainees) or better accommodating non-modifiable factors (for example, extending training periods and reducing intensity to facilitate recovery and adaptation) are suggested, with focus on groups at increased risk identified in this review.

## 1. Introduction

A fracture, being an acquired rupture or break of bone, is a significant and debilitating injury commonly seen among athletes and military personnel [1]. The aetiology of fractures can be varied, and they can occur as a result of an acute, high impact, traumatic event, or as a result of repetitive, sub-maximal overloading causing microtrauma, as is the case for stress or fatigue fracture [2,3]. The diagnosis of stress fractures typically involves a thorough patient history and physical examination. During a patient history, patients will usually describe a sudden increase in physical activity or load, without adequate rest or time for the bone to adapt to stresses and build tolerance [4,5]. Additional common presentations involve complaints of tenderness on palpation of bony structures, and localised pain that tends to worsen as activity increases, and eases to an ache during rest. Oedema and erythema may also be present. Stress fractures are generally confirmed through radiographs (X-ray), Magnetic Resonance Image (MRI), or bone scintigraphy (bone scans), with the latter considered the gold standard for stress fracture diagnosis [6].

Although stress fractures occur in the general population, they are more common among military populations due to the high physical loads their bodies are subjected to and the high-risk activities and substantial physical stresses to which they are exposed [3,4,7,8]. Basic training ranges from an eight to 32-week continuous training period, involving demanding tasks, such as running, jumping, marching, throwing or carrying various loads, over different terrains, and that can place high demands on the body [2,7,9]. As such, without adequate rest during training, it is not surprising that stress fractures are among the leading causes of injury for military personnel when undergoing basic training [7,10], as a mismatch can occur between bone breakdown (resorption from osteoclasts) and bone synthesis (regeneration from osteoblasts), favouring bone resorption [5,11]. 

A systematic review by Jones et al. [4] reported a period prevalence rate of stress fractures during 8-week Army basic combat training between 0.9% and 5.2% for males, and between 3.4% and 21.0 % for females. Furthermore, Jones et al. [4] reported the period prevalence rate of stress fractures during 12-week Marine basic training to be between 0.8% and 4.0% in males, and 3.0% and 5.7% in females [4]. In a large cohort of basic trainees in the US Army, Knapik et al. found an incidence rate of 19.3 (male) and 79.9 (female) cases of stress fractures per 1000 recruits within the 10 week period of basic training. Noting the relatively high rates of these injuries, injured personnel can take between 10–47 weeks to return to full training following stress fractures [3]. Not only does the occurrence of stress fractures place burden on the affected personnel, but it also results in additional costs to the organisation associated with prolonged rehabilitation, medical treatment, longer training periods, and time to return to military work. The costs associated with lower extremity stress fractures in trainees in the US Airforce has been quoted as exceeding USD $4.8 million per year [12].

Risk factors for stress fractures have been well documented in the literature and have been grouped into modifiable and non-modifiable factors, in an attempt to identify risks that may be mitigated [13]. Modifiable factors include: level of fitness [8,14,15,16,17,18], muscle strength [19], menstrual activity [8,15,18], and pretraining level of fitness/activity [8,14,16,20,21,22]. Non-modifiable factors that have been investigated include: age [15,16], sex [15,23,24], race/ethnicity [3,25], genotype [21], some kinanthropometric attributes [1,9,15,20,26], and previous history of injury to the fracture site [15,21,27].

Although there are many studies investigating risk factors for stress fractures in military settings, findings are often inconclusive. Further, previous studies have typically focused on identifying the modifiable risk factors, since those can be directly changed [4]. In contrast, to the authors’ best knowledge, there are no systematic reviews targeting non-modifiable risk factors for stress fractures in military populations. If non-modifiable factors can be identified and understood, then attention can focus on potential ways to address these factors indirectly (e.g., through accommodation for them), so as to mitigate their impacts and reduce stress fracture risk in military trainees. Therefore, the primary aim of this systematic review was to identify, critically review, and synthesise the findings of recent studies investigating non-modifiable risk factors for the development of stress fractures in military personnel undergoing training. 

## 2. Materials and Methods

### 2.1. Protocol

This literature review was guided by the Preferred Reporting Items for Systematic review and Meta-Analysis Protocols (PRISMA-P) [28]. The protocol for the overarching systematic review on fractures in occupational settings, of which this review formed one part, was registered in the Open Science Framework on 27 July 2020 (accessible at https://osf.io/n5jyx).

### 2.2. Information Sources and Search

Three databases known to publish studies relevant to this review, PubMed, Elton B. Stephens Company (with focus on SPORTDiscus and Cumulative Index of Nursing and Allied Health Literature (CINAHL)) and ProQuest (with focus on Military, Nursing & Allied Health, Public Health, and Health & Medical Collection), were systematically searched using a search string similar to the one displayed in Table 1 for PubMed. The reference lists of included articles were also manually searched to identify additional articles for inclusion.

### 2.3. Eligibility Criteria, Screening and Selection

The criteria adopted for study inclusion in this review were: (a) the study design was quantitative; (b) the study reported original research conducted in humans 16 years or older, or data for these groups were extractable; (c) the study was published in English, Portuguese, French, Italian or Spanish languages; (d) the study was published within the last 20 years; (e) the study investigated risk factors for stress fractures (as a subset of fractures) in any form of military training (as a subset of physically demanding occupations); and (f) the study included information on diagnostic procedures used to confirm stress fracture in the methodology. The criteria adopted for study exclusion were: (a) unable to access full text through search database or library sources; (b) unable to discern whether fractures were traumatic or stress fracture; (c) investigated only modifiable risk factors; (d) non-peer-reviewed; (e) non-military population; (f) military personnel not undertaking training; (g) investigated pharmacological interventions/ergonomic aids; (h) population comprised of individuals with a pre-existing medical condition; (i) published abstracts; and (j) qualitative studies.

The references identified in the search were exported from the above-listed databases to Endnote (version X9, Clarivate Analytics, Philadelphia, United States). Titles and abstracts of the articles were screened. Duplicates and articles that were clearly not relevant to the review, for example, studies investigating osteoporosis in post-menopausal women, were removed. This process was completed by two reviewers (G.L. and P.W.) separately and cross-referenced to identify agreement and differences, the latter of which were discussed to arrive at a decision regarding potential eligibility. Where consensus could not be reached, a third reviewer adjudicated (R.O.). Two reviewers (E.C. and V.S.) screened studies in included languages other than English (Table 1). Full-text copies of the remaining studies were obtained and subjected to assessment against the eligibility criteria by each of two authors (G.L. and P.W.) independently. Any disagreements between the two reviewers (G.L. and P.W.) regarding eligibility of full-text articles were resolved through discussion or in consultation with the third reviewer (R.O.) where needed. Excluded studies were removed, with reasons recorded. The results of the search, screening and selection processes were documented in a PRISMA flow diagram [29].

### 2.4. Methodological Quality Assessment

Studies that were included following the selection processes underwent a process of critical appraisal by two reviewers (G.L. and P.W.) to assess their methodological quality. The Critical Appraisal Skills Program (CASP) toolkit [30,31] was used to assess and appraise cohort [30] and case-control studies [31], whereas the AXIS [32] tool was used to appraise cross-sectional studies. The AXIS tool was used because the CASP toolkit does not have a tool to appraise cross-sectional studies. The CASP cohort study checklist [30] includes 12 questions that are broken down into three core sections: (1) validity of the study results based on methodological considerations, (2) what the results entail, and (3) whether the results will be useful in the context in which they will be applied. The CASP case-control study checklist [31] includes 11 questions that relate to screening and study design quality, validity and relevance. The AXIS [32] is a 20-question checklist containing 11 questions that assess the objectives and methods of the study, seven questions relating to the study’s results and discussion, and two questions relating to ethics.

Questions in each tool were rated on a binary scale, with 1 point awarded for questions that could be answered ‘yes’ and 0 points awarded for those that were answered ‘no’ or were indeterminable. An exception to this method was question 19 in the AXIS tool, where a ‘no’ answer was awarded 1 point, since answering ‘yes’ to that question affirms that there are funding sources or conflicts of interest that may affect the authors’ interpretation of results. Questions 7–9 on all the CASP tools were grouped together as one answer, since they were related and questions 7 and 8 were not answerable with a ‘yes’ or ‘no’ and as such could not be separately scored. The CASP case-control study checklist [29] was therefore graded out of nine possible points. Question 12 of the CASP cohort study checklist [33] could not be answered numerically; therefore, that checklist was also scored out of a total of 9 points. Cross-sectional studies appraised using the AXIS tool [30] were graded out of 20 points. Scores from each appraisal, with the CASP or AXIS tools, were converted to a percentage score to derive the final scores from the tools by dividing the assessed score by the maximum possible score and multiplying the result by 100. Percentage scores were then graded using a quality rating, from “very low” to “high”, where ≤20 = “very low”, 21–40 = “low”, 41–60 = “moderate”, 61–80 = “good”, and ≥81 = “high”. To ensure validity of scoring, two authors (G.L. & P.W.) independently assessed each of the studies and then discussed their assessments and resolved any disagreements through consensus or in consultation with a third reviewer (R.O.). The level of agreement between their initial appraisals was determined via a kappa analysis conducted by the third reviewer (R.O.).

### 2.5. Data Extraction and Synthesis

Following critical appraisal, relevant data were systematically extracted and tabulated from the included studies. Extracted data included: study title, year, authors, study type, population characteristics, information about the diagnostic process for stress fractures, methodological quality assessment score, and training details when available. The magnitudes of associations between non-modifiable risk factors and the risk of developing stress fractures [Risk Ratios (RR), Odds Ratios (OR) and 95% Confidence Intervals (CI)] and any related *p*-values were also extracted. Following data compilation and critical appraisal, key findings from included studies were analysed in a critical narrative synthesis.

## 3. Results

**Figure 1:**
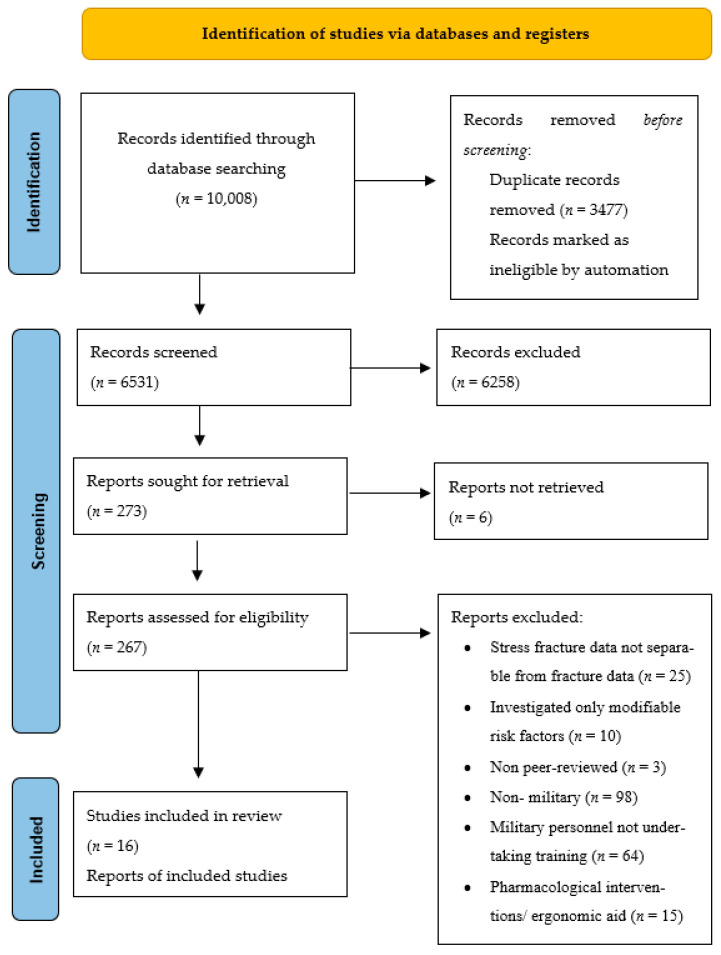
PRISMA 2020 flow diagram for new systematic reviews, which included searches of databases and registers only [34].

The systematic search conducted in Jan 2021 resulted in identification of a total of 10,008 articles. From these, 3477 duplicates were removed. The remaining 6531 articles were then screened against the eligibility criteria by title and abstract to exclude those clearly not relevant to the review topic. Following this, full texts of 273 articles of potential relevance were retrieved and 257 were excluded based on the eligibility criteria, as detailed in Figure 1. Once the screening and selection processes were complete, 16 articles remained to inform the review. The results of the search, screening and selection processes are documented in the PRISMA flow diagram shown in Figure 1 [28].

The mean ± standard deviation score for methodological quality of the included studies was 79.63 ± 9.67%, with the lowest score 64% [19] and highest score 100% [3]. The level of agreement between raters in critical appraisal scores reflected ‘almost perfect agreement’, as measured by the Cohen’s Kappa (k = 0.835) [32].

Of the studies informing this review, 14 were cohort studies [1,3,8,9,14,15,18,19,21,22,34,35,36,37], one was a case-control study [16] and one was a cross-sectional study [38] (Table 2). Stress fractures in Army [3,19,22,34,38] and Navy or Marine [1,3,10,18,20] trainees were reported in five studies each, while six studies [14,15,21,27,35,36] reported on stress fractures in military recruits without specifying service branch. Five studies [8,18,19,22,34] involved female trainees only, five [14,16,21,35,36] male trainees only, three [3,15,38] both female and male trainees, and three [1,9,27] did not specify the sex of trainees. The study type, participant demographic information, methods used to identify stress fracture cases, and quality rating for each included study are outlined in Table 2.

All studies reported on lower limb stress fractures, with three [9,18,21] reporting on tibial stress fractures, three [18,21,35] on stress fractures relating to the femur and pelvis and two [18,21] on metatarsal stress fractures. Twelve [1,3,8,14,15,16,19,22,27,34,36,38] did not specify a particular bone and investigated lower limb stress fractures in general. The specific non-modifiable risk factors investigated in the included studies are detailed in Table 3. 

Of the studies reporting on age as a non-modifiable risk factor, four studies [3,16,22,34] identified advancing age to be associated with a significantly increased risk of stress fractures in trainees. One study [1] found increasing age to be associated with reducing risk of 3rd metatarsal stress fracture risk. The remaining four studies [8,21,35,36] reported no significant associations between age and stress fracture risk. The ages of participants involved in this review ranged from 16 to 33 years (Table 4). 

Six of the studies reported on race. Three studies [3,34,38] reported that trainees of ‘black’ race were at lower risk of developing stress fractures than trainees of other races. Three studies found no significant association between race and stress fracture risk [8,18,22] (Table 4).

Of the studies reporting on prior injuries, two [21,27] found trainees who had ex-perienced a previous stress fracture were at an increased risk of developing another stress fracture, while conflicting results from one [18] paper found a history of prior stress fracture injury was associated with a slightly lower, although non-significant risk of developing another stress fracture. One study [8] found no significant association between previous stress fracture and stress fracture risk. Three studies investigated associations between prior self-reported musculoskeletal injury/ disease and stress fracture risk. All three studies [8,14,35] found no significant association between previous musculoskeletal injury and stress fracture risk (Table 4). 

The four studies reporting on the relationship between height and stress fracture risk did not identify height of trainees as a significant risk factor for stress fractures [18,19,21,36]. Average H heights of trainees in these studies ranged from 16,157.26 ± 6.5 cm [18] to 178.7 cm [36]. Studies that investigated height were either conducted in all female populations [18,19], all male populations [21] or included both sexes [36] (Table 4). Given that generally females are shorter than males and the number of studies which reported on females, this may have brought the average height down and may not be applicable for those who are taller than 179 cm.

All three studies investigating associations between menstrual dysfunction and risk of developing stress fractures [8,15,18] identified an increased risk of stress fractures in trainees reporting menstrual dysfunction. Cosman and colleagues [15] reported an association between increased stress fracture risk and a shorter time from menarche. Schaffer and colleagues [18] reported menstrual dysfunction (no menses during the past year or secondary amenorrhea) to be associated with increased risk of stress fractures in trainees. Raul and colleagues [8] reported a history of secondary amenorrhea during the year prior to basic training to be linked to an increased risk of stress fractures (Table 4).

Two studies looked at specific non-modifiable kinathropometric attributes and stress fracture risk. Nunns and colleagues [9] reported a significantly greater risk of tibial stress fractures in trainees with smaller bimalleolar width (*p* < 0.01), reduced tibial rotation (*p* < 0.05) and higher peak heel pressure (*p* < 0.001). Cosman and colleagues [15,22] reported statistically significant greater risk of stress fractures in males with lower cortical tibial dimensions and tibial bone mineral content (MBC), and in both males and females with lower femoral neck diameter (Table 4).

The remaining factors, sex and genotype, were each investigated in a single study (Table 3). Knapik and colleagues found female trainees to be at increased risk for stress fractures during military training when compared to males [3]. Zhao and colleagues [21] found stress fracture risk was significantly higher in individuals who exhibited the presence of the single nucleotide polymorphism, rs143383, in the GDF5 gene, which the authors noted ‘plays an important role in both intramembrane and endochondral bone formation during fracture healing’ (Table 4).

## 4. Discussion

The primary aim of this review was to identify, critically review, and synthesise the findings of recent studies investigating non-modifiable factors and their associations with the development of stress fractures in military personnel undergoing training. The overall methodological quality of the 16 articles identified for inclusion in this review ranged from good to high. Several non-modifiable risk factors for stress fractures in military personnel undergoing training were identified in this systematic review. These include increasing age, race other than black race, prior stress fracture, prior musculoskeletal injury, menstrual dysfunction, and specific kinathropometric attributes including smaller bimalleolar width, reduced tibial rotation, higher peak heel pressure, lower cortical tibial dimensions and tibial bone mineral content. Other risk factors which may be associated with stress fracture include both increasing age, race other than black race, Trainee height was also investigated but found to have no relationship to stress fracture risk in trainees. Furthermore, although each was only investigated in one single study [3,21], female sex and specific genotypes (single nucleotide polymorphism, rs143383, in the GDF5 gene) were found to increase risk for stress fractures in trainees. 

Four of the nine included studies that investigated age as a risk factor found a dose-response relationship between increasing age and risk of stress fractures in trainees [3,16,22,34]. The remaining studies identified little or negligible association between increased age and stress fracture risk. Of note, Knapik and colleagues [3] found a 2.29 (95% CI 2.09 to 2.51) and 3.50 (95% CI 3.20 to 3.82) fold increased risk of developing stress fractures in males and females, respectively, completing basic combat training when age increased over 30 years. Similarly, Cowan and colleagues [22] found a 3.07 (95% CI 1.81, 5.19) fold increased risk in female trainees over the age of 25 years. Older age has been consistently identified as a risk factor for stress fractures during military training among the literature when comparing age ranges of 17–19 vs. 21–29 [13], and 19–23 vs. 24–32 [16]. One previous study [39] found stress fracture risk decreased as age increased, however, the number of recruits over 19 years old was only 26, with the sample size being 783. Previous literature [40] found that increasing age over 19 years increased the risk for stress fractures during military training, and suggested the increased risk may be due to decreasing bone density, however, DXA measurements have disproven this argument [13]. Therefore, plausible explanations as to why increasing age may or may not be a risk factor are unknown and further re-search needs to be undertaken. Of note, the older ages referred to in these studies are in relation to the trainee population norms (e.g., 19–23 vs. 24–32) and, as such, these age ranges are still relatively young when considered against population norms. 

In regard to race, the volume of evidence suggests that black trainees may have a decreased risk of stress fractures during training when compared to all other racial/ethnic groups. This finding has been consistent among the literature [13,25,38]. When compared to other races/ethnic groups, black soldiers have been found to have higher average bone mineral density and thicker cortical bone mass [3,38]. Furthermore, black soldiers may have a slower age-adjusted yearly rate of decrease in bone mineral density [41]. Ettinger and colleagues [42], found that differences in bone mineral density seen among black soldiers, when compared to all other racial/ ethnic groups, could not be explained by clinical and kinathropometric attributes such as lifestyle factors, sun exposure, biochemical bone markers, dietary history, body composition and other factors. It is therefore worthy of further investigation as to why black soldiers appear to have a decreased risk of stress fractures during military training, when compared to other racial/ ethnic groups and also why these other groups are at a greater risk. 

Two of the three studies [21,27] reporting on prior stress fractures suggests that previous stress fractures significantly increase the risk of subsequent stress fractures during training, whereas one study found previous stress fracture to slightly reduce the risk of subsequent stress fracture, although insignificant. The majority of the studies on previous musculoskeletal injury history on risk of future stress fractures show a slight increase in risk, although insignificant. These findings are consistent with previous research which has found an increased risk of lower extremity injury after previous injury in both athletic and tactical personnel [27,43,44]. Following lower extremity injury, structural changes among soft tissue occur, along with alterations in kinematics, proprioception, and strength [43]. These changes alter motor control and function [43], thus leaving individuals more susceptible to subsequent injury. Furthermore, the risk is increased for subsequent injury in any anatomical location, not only the same location as previous injury [44]. It has been well documented that a previous stress fracture increases the risk of a subsequent stress fracture [8]. Therefore, appropriate rehabilitation of any past or current injuries is vital to return to training as is an increased tolerance to the loads that their bodies will be subjected to during training. A well designed, comprehensive and structured rehabilitation program, utilising regional interdependence in order to reduce risk of subsequent injury throughout the kinetic chain can be suggested [43].

The results suggest female recruits may be at increased risk for stress fractures if they have a shorter time from menarche (time of menarche relative to when the stress fracture occurred), no menses during the past year, or a history of secondary amenorrhea. Previous literature has suggested stress fracture occurrence in this population may be related to a subsequent low bone mineral density due to the irregular menses [2]. Worthy of noting, is the increased risk of stress fractures seen in females as a sex, reported previously among the literature [3,38,45]. Although only one study in this systematic review investigated sex as a risk factor, the results supported previous literature that suggests female sex is at increased risk for stress fractures during military training, when compared to males [13,46]. In this review, Knapik and colleagues [3] found a 3.85 (95% CI 3.66–5.05) increased risk in female trainees when compared to male trainees. Previous research has suggested this may be due to multiple factors including but not limited to absolute aerobic capacity; body fat mass; the female athlete triad (oligomenorrhea or amenorrhea, osteoporosis and eating disorders with energy deficit); muscle strength; mean stature and bone characteristics such as long bone length and cortical thickness [3,12,47]. 

Only two studies investigated the influence kinathropometric attributes have on stress fracture risk in military personnel undergoing training. Findings indicate a rela-tionship between bone size (reduced bimalleolar width, and lower femoral neck diameter in both males and females; and lower cortical tibial dimensions in males), may significantly increase the risk of stress fractures in trainees [9,15]. However, as only one study investigated each kinathropometric attributes more research is needed to determine whether certain kinathropometric attributes such as bimalleolar width and femoral neck diameter can influence stress fracture risk in military personnel undergoing training. 

Several non-modifiable risk factors identified in this systematic review, such as advancing age; prior stress fracture history; menstrual related factors and sex, while non-modifiable can be considered in the basic training of military personnel. For example, while trainees have no influence on their advancing age or previous injury, it may be beneficial for older trainees or those who have suffered an injury to ensure they are well prepared physically to undergo basic training. Likewise, the training undertaken should consider the age range of those attending training and those who are recovering from injuries by employing concepts like ability-based training (as opposed to a one-size fits all approach) may help limit the potential for excessive loading [48]. While female trainees cannot directly change the time from menarche, the number of menses during previous years, or their history of amenorrhea or secondary amenorrhea, several factors can be modified to reduce their risk of menstrual disorders. Beyond ensuring female trainees have prepared their bodies physically for the demands of training, trainees could address factors such as nutrition and body fat mass, both of which have been identified as modifiable risk factors for stress fractures in military personnel undergoing training [13]. Orr and colleagues [49] recently re-viewed injury risk factors associated with load carriage in female military personnel. From their review, the influence of the female athlete triad is worthy of noting, with regard to the association between increased stress fracture risk and a combination of menstrual dysfunction, low energy availability and low bone mineral density. Since female trainees may not be able to directly change their previous menstrual function, strategies to reduce the risk of the female athlete triad can be suggested. Such Strategies proposed previously to address this have included include appropriate education regarding the triad and the potential negative effects associated with it [50]; optimizing energy availability with special attention paid to vitamin D and calcium intake [50]; and appropriate, well-structured and progressive conditioning programs prior to military training with the consideration of load management to allow appropriate time for recovery of bone stress [49].

As identified in a 2021 systematic review by Wood and colleagues [51] poor prior exercise history, low current fitness test results, and higher BMI are among the top identified modifiable risk factors for stress fractures in military personnel undergoing training. Strategies to mitigate these factors may indirectly and positively impact on non-modifiable risk factors. Studies have shown that the implementation of resistance, aerobic and plyometric training can increase muscle strength, hypertrophy and endurance; increase peak bone mass; build soft tissue tolerance and load capacity; and thus, reduce the risk of stress fractures in military personnel [52,53,54]. Furthermore, resistance training has shown to have positive effects on biomarkers of bone formation by creating an osteogenic stimulus [53,54,55]. This is of note given that both fitness and bone density are known to decrease with age [13] and female trainees are generally less fit than male trainees [56] and tend towards higher fat mass and BMI [49]. 

As the non-modifiable risk factors for stress fractures in military personnel un-dergoing training have been identified, focus can now be shifted to identify ways to mitigate factors that can be controlled in the populations identified. For example, since previous stress fracture increases the risk of subsequent stress fracture, research on appropriate reconditioning requirements prior to basic training can be suggested. This may include looking at factors such as volume, intensity and type of training, acute to chronic workload ratios and frequency of training before basic training. Furthermore, finding ways to quantify the modifiable risk factors identified in this study to help provide guidelines to recruits, staff and clinicians to help decrease the risk of stress fractures during training is a potential topic for further investigation.

### Strengths and Limitations 

This systematic review did not come without limitations. Although the studies were of good quality, 3/16 were retrospective in design, which may have subjected them to selection and recall bias. Furthermore, the heterogeneity of the studies was high, which did not enable a meta-analysis to be conducted. Through the rigorous selection criteria, cross referencing and marking through multiple authors, author bias was reduced. According to the “new proposed level of evidence” by Murad and colleagues [57] cohort studies are level 3 evidence, while case control and cross-sectional are level 4 evidence. Fourteen of our studies were therefore of level 3 evidence, and two were of level 4. Although randomised control trials are the higher level of evidence for identifying risk factors and prognostic values, cohort, cross-sectional and case-control studies are the preferred method of study design [57]. 

## 5. Conclusions

The main non-modifiable risk factors for stress fractures in military trainees iden-tified were advancing age, race other than black race, prior stress fracture, the female sex, and menstrual dysfunction (no menses during the past year or secondary amen-orrhea), while advancing age and race other than black race may be risk factors. To reduce the incidence of stress fractures in military trainees, efforts should be made to optimise modifiable risk factors (e.g., fitness levels) in personnel who possess non-modifiable factors identified by this review that are associated with increased risk of stress fracture development (e.g., older age). 

## Figures and Tables

**Table 1:** Search terms used in PubMed.

Database	Search Terms	Filters
PubMed	(((risk[Title/Abstract] OR predict * [Title/Abstract] OR prevalence[Title/Abstract] OR incidence[Title/Abstract] OR caus * [Title/Abstract] OR etiol * [Title/Abstract] OR frequenc * [Title/Abstract] OR rate * [Title/Abstract] OR mediat * [Title/Abstract] OR exposure * [Title/Abstract] OR likelihood[Title/Abstract] OR probability[Title/Abstract] OR factor[Title/Abstract] OR factors[Title/Abstract] OR hazard[Title/Abstract] OR hazards[Title/Abstract] OR predisposing[Title/Abstract])) AND ((work * [Title/Abstract] OR occupation * [Title/Abstract] OR profession * [Title/Abstract] OR trade[Title/Abstract] OR employ * [Title/Abstract] OR military[Title/Abstract] OR Defence[Title/Abstract] OR Defense[Title/Abstract] OR airforce[Title/Abstract] OR “air force”[Title/Abstract] OR army[Title/Abstract] OR navy[Title/Abstract] OR recruit[Title/Abstract] OR soldier * [Title/Abstract] OR marines[Title/Abstract] OR “Military Personnel”[Title/Abstract]))) AND ((Fracture * [Title/Abstract]))	Humans, English, Spanish, Portuguese, Italian, French, Adolescent 13−18 years, Adults 19+ years, 2000−2020

* = truncation for the search.

**Table 2:** Study characteristics.

Reference	Study Type	Participants	Methods (Stress Fracture Diagnosis)	Occupational Training Program	Methodological Quality Rating (%)
Cosman et al. (2013) [15]	Prospective cohort	Total: *n* = 891	Orthopedist assessment X-ray	United States Military Academy Cadet Training	High (91)
		U.S. Military Academy cadets			
		Male: *n* = 755			
		Mean ± SD Age: 18.7 ± 0.8 years;	CT scan or MRI		
		Female: *n* = 136			
		Mean ± SD Age: 18.4 ± 0.8 years			
Cowan et al. (2012) [22]	Prospective cohort	Total: *n* = 1568	At least two encounters with the same diagnosis, using ICD-9 codes 733.93 (tibia or fibula), 733.94 (metatarsals) and 733.95 (other bone)	Army Basic Training	Good (73)
		Females entering the U.S. Army			
Dixon et al. (2018) [1]	Prospective cohort	Total: *n* = 1065	Not detailed	32 Week Royal Marine Training Program	High (82)
		UK Royal Marine recruits			
Knapik et al. (2012) [3]	Retrospective cohort	Total: *n* = 583,651	ICD-9 codes	10 weeks of basic training	High (100)
		U.S. military recruits from databases of the Armed Forces Health Surveillance			
		Females: *n* = 10,706			
		Males: *n*= 475,745			
Knapik et al. (2018) [38]	Cross-sectional	Total: *n* = 583,651	ICD-9 codes 733.1–733.19 and 733.93–733.98	Army Basic Training	High (85)
		U.S. military recruits			
		Males: 475,745			
		Females: 107,906			
Kucera et al. (2016) [27]	Prospective cohort	Total: *n* = 9811 U.S. military cadets	ICD-9 codes	2-month U.S. cadet Basic Training	Good (73)
Lappe et al. (2001) [34]	Prospective cohort	Total: *n* = 3758 female U.S. military recruits	Clinical assessment X-ray or CT scan	8-week U.S. Basic Military Training including:	High (82)
				(1) March 225 km on gravel roads carrying a 10 kg pack and rifle	
				(2) Run 135 km on asphalt roads	
				(3) Approximately 1 h/day of physical training	
				(4) Traverse an ‘agility course’ four times during the last 4 weeks	
Nunns et al. (2015) [9]	Prospective cohort	Total: *n* = 1065 UK Royal Marine recruits	Medical examination	Royal Marine 32-week training program	Good (73)
		Logistic regression analysis to assess potential risk factors focused on subsamples of recruits who sustained a tibial stress fracture (*n* = 10) and an injury-free group (*n* = 120)	MRI		
Pihlajamäki et al. (2019) [35]	Prospective cohort	Total: *n* = 4029 male Finnish military recruits	ICD-9 or ICD-10 diagnosis codes indicating stress fracture	8 weeks of basic military training including:	High (82)
				-17 h per week of combat skills, marching and other physically demanding training	
				-Carrying heavy loads	
Pihlajamaki et al. (2006) [37]	Retrospective cohort	Total: *n* = 4029 male Finnish military recruits	Clinical examination	8 weeks of basic military training including:	Good (73)
			X-ray	-17 h per week of combat skills, marching and other physically demanding training	
			MRI or CT scan	-Carrying heavy loads	
Rauh et al. (2006) [8]	Prospective cohort	Total: *n* = 824 female U.S. Marine Corps recruits	Clinical examination	Marine Corps Recruit depot basic training	High (91)
			X-ray		
			CT scan		
Schaffer et al. (2006) [18]	Prospective cohort	Total: *n* = 2962 female U.S. Marine Corps recruits	Clinical presentation with diagnostic imaging (X-ray, bone scan or both)	13-week U.S Marine Corps basic training	High (82)
		Aged 17–33 years			
Sanchez-Santos et al. (2017) [16]	Case-control	Total: *n* = 1082 UK Royal Marine recruits aged 16–33 years, including 86 cases with stress fractures	Clinical examination	32 weeks of Royal Marine training	Good (77)
			X-ray		
			CT scan		
Scheinowitz et al. (2017) [19]	Prospective cohort	Total: *n* = 226 female Israeli military recruits	Clinical examination	16-month combat Army Basic Training program in the Israeli Defense Forces	Good (64)
			X-ray		
			CT scan		
Sormaala et al. (2006) [36]	Retrospective cohort	Total: *n* = 30 male Finnish military recruits, age range 18–26 years	Physical examination by orthopaedic surgeon	Military Training Program	High (82)
			X-ray		
			CT scan		
Zhao et al. (2016) [21]	Prospective cohort	Total: *n* = 1398 male Chinese infantry recruits	Clinical examination	8-week training program including marching, running, training exercises and stationary standing procedures	High (82)
			X-ray		

**Table 3:** Non-modifiable factors investigated for their associations with stress fracture risk.

Variable	Number of Studies	References
Age	9	[1,3,8,16,21,22,34,35,36]
Race	6	[3,18,22,34,38]
History of stress fracture	4	[8,18,21,27]
Height	4	[18,19,21,36]
History of musculoskeletal injury	4	[8,14,18,35]
Menstrual dysfunction	3	[8,15,18]
Kinathropometric attributes	2	[9,15]
Sex	1	[3]
Genotype	1	[21]

**Table 4:** Non-modifiable risk factors and associated relative risks or odds of stress fractures.

Study	Type of Fracture	Non-Modifiable Risk Factor		Key Findings		*p*-Value
Cosman et al. (2013). [15]	Stress fracture	Each additional year from menarche		♂ OR (95% CI)	-	
				♀ OR (95% CI)	1.44 (1.19, 1.783)	
		Diameter of femoral neck (mm) (each mm decrease)		♂ OR (95% CI)	1.35 (1.01, 1.81)	
				♀ OR (95% CI)	1.16 (1.01, 1.33)	
		Tibial BMC (mg) (each 10 mg decrease)		♂ OR (95% CI)	1.11 (1.03, 1.20)	
				♀ OR (95% CI)	1.03 (0.92, 1.16)	
		Tibial cortex cross-sectional area (each mm^2^ decrease)		♂ OR (95% CI)	1.12 (1.03, 1.23)	
				♀ OR (95% CI)	1.01 (0.89, 1.15)	
Cowan et al. (2012) [22]	Stress fracture	Age (years)	18–19 (reference)	OR (95% CI)	1.00	
			20–24		2.06 (1.32, 3.20)	
			≥25		3.07 (1.81, 5.19)	
		Race	White (reference)	OR (95% CI)	1.00	
			Black		0.68 (0.42, 1.12)	
			Other		0.97 (0.57, 1.66)	
Dixon et al. (2018) [1]	2nd metatarsal fracture	Age (each additional year)		RRR (95% CI)	1.06 (0.85–1.32)	
	3rd metatarsal fracture	Age (each additional year)			0.78 (0.61–0.99)	
Knapik et al. (2012) [3]	Stress fracture	Age (years)	<20 years (reference)	♂ OR (95% CI)	1.00	
				♀ OR (95% CI)	1.00	
			20–24	♂ OR (95% CI)	1.41 (1.34–1.48)	
				♀ OR (95% CI)	1.47 (1.40–1.54)	
			25–29	♂ OR (95% CI)	1.80 (1.67–1.93)	
				♀ OR (95% CI)	2.33 (2.19–2.49)	
			≥30	♂ OR (95% CI)	2.29 (2.09–2.51)	
				♀ OR (95% CI)	3.50 (3.20–3.82)	
		Race/ethnicity	White	♂ OR (95% CI)	1.54 (1.46–1.63)	
				♀ OR (95% CI)	1.74 (1.62–1.87)	
			Black (reference)	♂ OR (95% CI)	1.00	
				♀ OR (95% CI)	1.00	
			Hispanic	♂ OR (95% CI)	1.40 (1.30–1.52)	
				♀ OR (95% CI)	1.58 (1.44–1.73)	
			Asian	♂ OR (95% CI)	1.23 (1.08–1.41)	
				♀ OR (95% CI)	1.29 (1.12–1.48)	
			American Indian	♂ OR (95% CI)	1.39 (1.16–1.65)	
				♀ OR (95% CI)	1.80 (1.46–2.21)	
			Other	♂ OR (95% CI)	1.78 (1.30–2.44)	
				♀ OR (95% CI)	2.08 (1.48–2.92)	
			Unknown	♂ OR (95% CI)	1.20 (0.98–1.46)	
				♀ OR (95% CI)	1.24 (0.99–1.55)	
		Sex	Male	♂ OR (95% CI)	1.00	
			Female	♀ OR (95% CI)	3.85 (3.66–5.05)	
Knapik et al. (2018) [38]		Race/ethnicity	Black (reference)	♂ OR (95% CI)	1.00	-
				♀ OR (95% CI)	1.00	-
			White	♂ OR (95% CI)	1.72 (1.60–1.85)	<0.01
				♀ OR (95% CI)	1.54 (1.46–1.63)	<0.01
			Hispanic	♂ OR (95% CI)	1.56 (1.43–1.71)	<0.01
				♀ OR (95% CI)	1.37 (1.27–1.48)	<0.01
			Asian	♂ OR (95% CI)	1.38 (1.20–1.59)	<0.01
				♀ OR (95% CI)	1.28 (1.12–1.46)	<0.01
			American Indian	♂ OR (95% CI)	1.75 (1.42–2.15)	<0.01
				♀ OR (95% CI)	1.37 (1.15–1.64)	<0.01
			Other	♂ OR (95% CI)	2.12 (1.52–2.98)	<0.01
				♀ OR (95% CI)	1.77 (1.29–2.43)	<0.01
			Unknown	♂ OR (95% CI)	1.31 (1.04–1.64)	0.02
				♀ OR (95% CI)	1.23 (1.01–1.51)	0.04
Kucera et al. (2016) [27]	No stress fracture history (reference)	Any history of injury to site		♂ OR (95% CI)	1.00	
				♀ OR (95% CI)	1.00	
		History of injury to site with activity limitation		♂ OR (95% CI)	1.00	
				♀ OR (95% CI)	1.00	
	Prior stress fracture	Any history of injury to site		♂ OR (95% CI)	3.58 (1.13–11.34)	
				♀ OR (95% CI)	17.03 (4.73–61.29)	
		History of injury to site with activity limitation		♂ OR (95% CI)	6.06 (3.02–12.14)	
				♀ OR (95% CI)	9.68 (3.91–23.95)	
Lappe et al. (2001) [34]		Age (for each additional year)		Adjusted RR (95% CI)	1.07 (1.04–1.1)	
		Race/ethnicity	White	Adjusted RR (95% CI)	1.17 (1.06–1.30)	
			Black (reference)		1.00	
			All other races		1.53 (1.02–2.28)	
Nunns et al. (2016) [9]	Tibial stress fracture	Bimalleolar width (mm) (for each 1 mm increase)		OR (95% CI)	0.73 (0.58–0.93)	
				Threshold for avoiding stress fracture	>74 mm	
		Peak heel pressure (N/cm^2^) (for each 1 N/cm^2^ increase)		OR (95% CI)	1.25 (1.07–1.46)	
				Threshold for avoiding stress fracture	<13 N/cm^2^	
		Tibial range of motion (°) (for each 1° increase)		OR (95% CI)	0.78 (0.63–0.96)	
				Threshold for avoiding stress fracture	>13°	
Pihlajamäki et al. (2019) [14]		Diseases of the musculoskeletal system	No disease history	IRR (95% CI)	1.00	
			Disease history		1.46 (0.57–3.70)	
Pihlajamaki et al. (2006) [35]		Diseases of the musculoskeletal system	No disease history	IRR (95% CI)	1.00	
			Disease history		1.36 (0.36–2.28)	
Rauh et al. (2006) [8]		Race/ethnicity	Black (reference)	OR (95% CI)	1.00	
			Caucasian		1.3 (0.6–2.7)	
			Hispanic		0.8 (0.3–2.2)	
			Asian		1.2 (0.2–5.9)	
			American Indian/Other		1.4 (0.3–6.7)	
		Age (years)	17–19 (reference)	OR (95% CI)	1.00	
			>20		1.7 (0.8–3.6)	
		History of lower extremity stress fracture	No (reference)	OR (95% CI)	1.00	
			Yes		2.1 (0.8–5.5)	
		History of lower extremity non-stress fracture injury	No (reference)	OR (95% CI)	1.00	
			Yes		1.1 (0.6–1.9)	
	Stress fracture	Secondary amenorrhea	No	Adjusted OR (95% CI)	1.00	
			Yes		2.7 (1.1–6.9)	
Sanchez-Santos et al. (2017) [16]		Age (years)	19–23	OR (95% CI)	1.66 (0.97–2.85)	0.66
			23–32		1.98 (1.07–3.55)	0.30
Schaffer et al. (2006) [18]	Overall stress fracture	Race/ethnicity	Black	OR (95% CI)	1.00	
			White		1.54 (0.9–2.5)	
			Hispanic		1.97 (1.1–3.7)	
			Asian		2.28 (0.9–5.6)	
			American Indian/other		1.10 (0.3–3.6)	
		Height (cm)	Shortest (≤157.26 cm)	OR (95% CI)	1.30 (0.8–2.1)	
			Mean (163.77 cm)		1.00	
			Tallest (≥170.29 cm)		1.28 (0.8–2.1)	
		History of stress fracture	No	OR (95% CI)	1.00	
			Yes		0.78 (0.2–2.5)	
		History of lower extremity injury	No	OR (95% CI)	1.00	
			Yes		0.77 (0.5–1.1)	
		Onset of menarche	≤15 years old	OR (95% CI)	1.00	
			16 years or older		1.29 (0.6–2.7)	
		Menses during past year	10–12	OR (95% CI)	1.00	
			1–9		0.77 (0.5–1.3)	
			None		5.64 (2.2–14.4)	
		Secondary amenorrhea during past year	No	OR (95% CI)	1.00	
			Yes		1.66 (0.8–3.4)	
	Pelvic or femoral stress fracture	Race/ethnicity	Black	OR (95% CI)	1.00	
			White		1.41 (0.7–2.9)	
			Hispanic		1.77 (0.7–4.4)	
			Asian		2.71 (0.8–9.0)	
			American Indian/other		1.50 (0.3–7.0)	
		Height (cm)	Shortest (≤157.26 cm)	OR (95% CI)	1.64 (0.9–3.1)	
			Mean (163.77 cm)		1.00	
			Tallest (≥170.29 cm)		1.40 (0.7–2.8)	
		History of stress fracture	No	OR (95% CI)	1.00	
			Yes		1.20 (0.3–4.9)	
		History of lower extremity injury	No	OR (95% CI)	1.00	
			Yes		0.71 (0.4–1.2)	
		Onset of menarche	≤15 years old	OR (95% CI)	1.00	
			16 years or older		0.34 (0.1–2.4)	
		Menses during past year	10–12	OR (95% CI)	1.00	
			1–9		1.25 (0.6–2.4)	
			None		8.54 (2.8–25.8)	
		Secondary amenorrhea during past year	No	OR (95% CI)	1.00	
			Yes		2.53 (1.1–6.0)	
Scheinowitz et al. (2017) [19]	Stress fracture	Mean ± SD Height (cm)			166 ± 6	0.006
	No stress fracture				162 ± 6	
Sormaala et al. (2006) [36]		Height (cm)			178	No significant differences in the average height of participants with or without stress fractures (*p* > 0.1)
		Age (years)			18–27	No significant differences in the average age of patients with or without stress fractures (*p* > 0.1)
Zhao et al. (2016) [21]	Stress fracture	Genotypes	Codominant	OR (95% CI)	1.76 (1.29–2.38)	*p* < 0.001
			TC			
			Dominant		2.91 (1.25–6.74)	*p* = 0.013
			CC			
			Recessive		1.83 (1.33–2.52)	*p* < 0.001
			CC þ TC			
		Mean ± SD Age (years)		OR (95% CI)	18.5 ± 1.4	NS (compared to no stress fracture)
		Mean ± SD Height (cm)			172.25 ± 5.67	NS (compared to no stress fracture)
		Prior fracture (*n* (%))			28 (14.8%)	*p* = 0.01 (compared to no stress fracture)
	No stress fracture	Mean ± SD Age (years)		OR (95% CI)	18.5 ± 1.8	
		Mean ± SD Height (cm)			171.78 ± 4.71	
		Prior fracture (*n* (%))			108 (8.9%)	

Legend: OR—odds ratio; RR—risk ratio; IRR—interrater reliability; NS—non-significant; ♂—female; ♀—male.

## Data Availability

Not applicable.
